# Phacoemulsification Induced Transient Swelling of Corneal Descemet’s Endothelium Complex Imaged with Ultra-High Resolution Optical Coherence Tomography

**DOI:** 10.1371/journal.pone.0080986

**Published:** 2013-11-28

**Authors:** Aizhu Tao, Zhao Chen, Yilei Shao, Jianhua Wang, Yune Zhao, Ping Lu, Fan Lu

**Affiliations:** 1 School of Ophthalmology and Optometry, Wenzhou Medical College, Wenzhou, Zhejiang, China; 2 Bascom Palmer Eye Institute, University of Miami, Miami, Florida, United States of America; Duke University, United States of America

## Abstract

**Purpose:**

Thickness changes of corneal sub-layers after phacoemulsification were investigated by spectral domain ultra-high resolution optical coherence tomography (UHR-OCT).

**Methods:**

The corneas (n = 26) of 26 age-related cataract surgery patients were studied. UHR-OCT was used to evaluate the thickness of Descemet’s Endothelium Complex (DEC), stroma, Bowman’s layer, epithelium, and full cornea at the center (CCT) before, one day after, and one week after surgery. Non-contact specular microscopy measured CCT, endothelial cell density, and morphology.

**Results:**

The DEC, stroma, Bowman’s layer, and epithelium were visualized by UHR-OCT. Before surgery, the DEC in all cases appeared as a translucent space between two smooth opaque lines. One day after surgery, the posterior corneal surfaces in half of the eyes were wavy and irregular. Compared to the baseline, one day after surgery the thickness increases of the DEC, stroma, and CCT were 4.3 ± 2.6 µm, 25.5 ± 24.9 µm, and 32.1 ± 26.6 µm, respectively (P < 0.001). The morphology of the DEC and the CCT recovered to baseline one week after surgery (P > 0.05), but endothelial cell density was 8.7% less than at baseline. There were no significant changes in Bowman’s layer and epithelium after the operation. The pre-operative DEC thickness was positively correlated with the decreased endothelial cell density at 1 day after surgery (r = 0.55, P = 0.003).

**Conclusions:**

The DEC showed edematous thickening and different degrees of morphological changes after phacoemulsification. The DEC deformation and corneal edema recovered by one week after surgery, which indicated recovery of endothelial function. UHR-OCT is a useful tool to evaluate function of the DEC after phacoemulsification. Pre-operative DEC thickness may indicate the integrity of the endothelium and could be used for predicting endothelial cell loss after phacoemulsification.

## Introduction

The evolution of phacoemulsification surgery has resulted in a trend towards improved visual acuity with optimal safety and minimum invasiveness [1]. However, transient corneal edema is a major factor delaying vision improvement during the early post-operative period [2]. Proper corneal hydration, required for the normal thickness and transparency of the cornea, is maintained by active electrolyte and fluid pumping by cells of the endothelium [3]. During normal aging, there is a decrease in the density of corneal endothelial cells that occurs simultaneously with the development of age-related cataract [4]. This loss of endothelial cells can be accelerated during cataract surgery and can result in corneal decompensation [5]. 

Several methods have been used to assess endothelial function post-operatively: specular microscopy and in vivo confocal microscopy for endothelial morphology, fluorophotometric measurement of corneal barrier functions, and pachymetry for central corneal thickness (CCT) [6-9]. However, some of these techniques require contact with the eye and none of them is able to document the thickness of the endothelium. With the improvement of axial resolution of optical coherence tomography (OCT), more detailed structural information of the cornea can be obtained [10]. Descemet’s membrane has been identified as the basement membrane of the endothelium [11], and we refer to Descemet’s membrane and the endothelium as the Descemet’s Endothelium Complex (DEC). With ultra-high resolution OCT (UHR-OCT), it is now possible to image the DEC [10,12]. The aim of this study was to determine thickness changes in the central DEC and other corneal sub-layers after phacoemulsification.

## Subjects and Methods

All patients were candidates for phacoemulsification surgery and intraocular lens implantation at the Eye Hospital, Wenzhou Medical College, Wenzhou, Zhejiang, China. The study was approved by the Wenzhou Medical College review board, and written informed consent was obtained from each patient. All patients were treated in accordance with the tenets of the Declaration of Helsinki. 

Age-related cataract was diagnosed on slit-lamp examination based on the opacity of the crystalline lens by the same examiner (ZC). For each patient, the cataract hardness was between II and IV according to the Emery and Little nuclear hardness classification [13]. Exclusion criteria included other current ocular diseases or systemic diseases. Cases were excluded if they had a history of ocular surgery or contact lens wear. 

### Procedure

All subjects were tested pre-operatively as well as one day and one week after surgery. The cataract was diagnosed bilaterally for each subject, but only one eye of each subject was randomly selected for study. The measurements were executed between 10:00 am and 5:00 pm. During each visit, non-contact UHR-OCT, specular microscopy, and tonometry were performed in the selected eye of each subject by the same veteran examiner (ZC). 

### Surgery

The surgical procedure was performed by the same veteran surgeon (YZ). An Infiniti phacoemulsificator (Alcon Laboratories, Fort Worth, TX, USA) was used. The surgical settings were shown in Table 1. The value of cumulative dissipated energy (CDE) was calculated as follows: CDE = (average ultrasound power × ultrasound time) + (torsional amplitude × torsional time × 0.4) [14]. All patients received proxymetacaine hydrochloride 0.5% for topical anesthesia at the beginning of the procedure. A standard temporal clear cornea incision was made, and a foldable intraocular lens was implanted in all patients (Table 2). Balanced salt solution (BSS) was used as the irrigating solution. Iviz (Sodium hyaluronate 1.0%, Zhengda Freda Pharmacy Limited Company, Shandong, China) was used in 21 patients and Duovisc (Alcon Laboratories, Fort Worth, TX, USA) was used in 5 patients as viscoelastic substances during surgery. The mean ± standard deviation effective phacoemulsification time was 46.0 ± 21.5 seconds. 

**Table 1:** Surgical settings.

	Group 1	Group 2
Number of Patients	n = 7	n = 19
System	Infiniti system (Alcon Laboratories, Inc.)	Infiniti system (Alcon Laboratories, Inc.)
Keratome	Diamond cutter 2.2 mm	Diamond cutter 3.0 mm
Implant System	Monarch® D cartridge	Monarch® C cartridge
Phaco Tip	45-degree, mini-flare, ABS, Kelman	45-degree, mini-flare, ABS, Kelman
MicroSmooth Sleeve	Micro-coaxial	Standard
Fluidic Management System	Intrepid FMS	Standard FMS
Phacoemulsification Setup		
Vacuum (mmHg)	360-400 linear	360-400 linear
Power (%)	Ozil continuous (torsional linear 50-100; phaco = 0)	Ozil continuous (torsional linear 50-100; phaco = 0)
Aspiration Rate	35.0 cc/min	35.0 cc/min
With IP software	Yes	Yes
Bottle Height (cm)	100	100

**Table 2:** Intraocular lenses implanted after phacoemulsification.

Lens	Number of Patients	Source
SN60WF	12	Alcon, Fort Worth, TX, USA
ADAPT-AO	5	Bausch & Lomb, Rochester, NY, USA
SN60T3	2	Alcon, Fort Worth, TX, USA
MI60	1	Bausch & Lomb, Rochester, NY, USA
SOFTEC HD	1	Lenstec, St. Petersburg, FL, USA
SN60AT	1	Alcon, Fort Worth, TX, USA
SOFTEC 1	1	Lenstec, St. Petersburg, FL, USA
XLSTABI-ZO	1	Carl Zeiss Meditec SAS, La Rochelle, France
SN6AD1	1	Alcon, Fort Worth, TX, USA
AKREOS ADAPT	1	Bausch & Lomb, Rochester, NY, USA

Duovisc was used when the lens nucleus was hard, or corneal endothelial cell density was less than 2,000 cells/mm^2^. Among the five patients for whom Duovisc was used, the lens nucleus of four subjects was graded III, and the nucleus of the other subject was graded IV. The mean phacoemulsification time in the Duovisc group was 58.8 sec, and 43.0 sec in the Iviz group. The bottle height was 100.0 cm. The mean phacoemulsification energy was 22.9 ± 8.9%. The mean size of corneal incision was 2.8 ± 0.4 mm. For seven patients, the corneal incision size was 2.2 mm, and for the other 19 patients it was 3.0 mm. No intracameral antibiotics or intracameral anesthesia was used. The surgical procedure was free of complications for all patients. A topical combination of steroids and antibiotics was given three times daily for 4 weeks as the post-operative treatment.

### Instrumentation

#### Spectral domain UHR-OCT

To detect the sub-layers of the cornea, a custom-built high speed, spectral domain UHR-OCT instrument was used for this study. It was similar to the device used in previously published studies [15,16]. A superluminescent diode light source (Broadlighter, T840-HP, Superlumdiodes Ltd, Moscow, Russia) with a full width at half maximum bandwidth at 100 nm, centered at 840 nm, was used to provide low coherence light. A telecentric light delivery system and video viewing system were co-axially aligned and mounted with a standard slit-lamp. The scan width was up to 15.0 mm and the scan depth was 3.0 mm in air. The A-line rate of the system was 24.0 kHz. The calibrated axial resolution of this device was ~3.0 μm in tissue with a refractive index of 1.389 [17]. Images captured by the camera were delivered to a computer workstation for display and processing.

**Figure 1:**
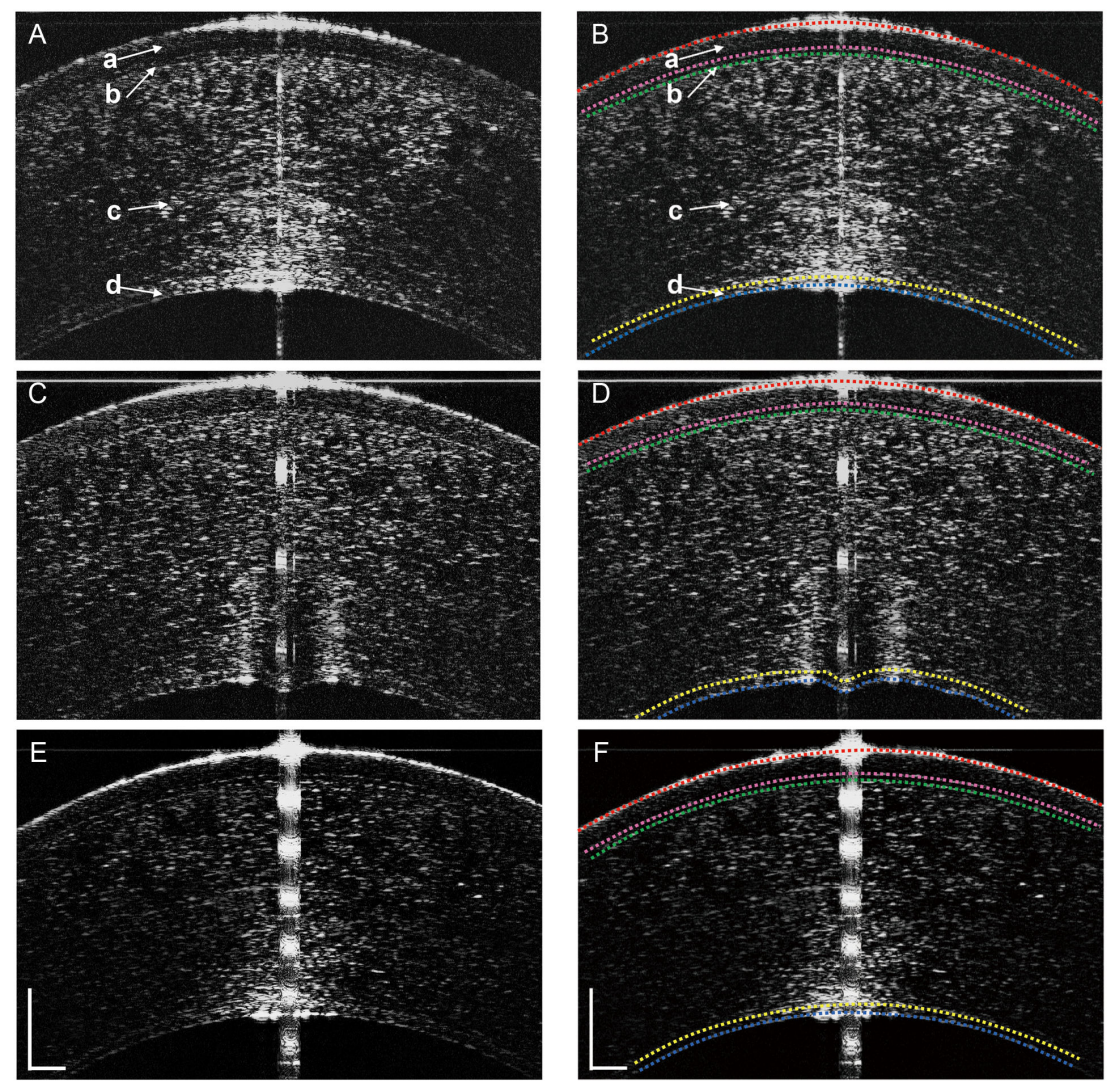
Representative UHR-OCT images of the central cornea before and after phacoemulsification surgery. Using custom developed software (J-OCT-1), the epithelium, Bowman’s layer, stroma, and Descemet’s Endothelium Complex (DEC) were identified semi-automatically as the tissue between the identified interfaces (colored dotted lines on the image). Raw (**A**) and semi-automatically segmented (**B**) images before surgery showed the epithelium (a), Bowman’s layer (b), stroma (c), and the DEC (d). At Day 1 after surgery the raw (**C**) and segmented (**D**) images showed swelling of the central cornea. The DEC appeared as a thickened band composed of two opaque lines separated by a translucent space. The anterior surface at the corneal epithelium was smooth while the posterior surface at the endothelium had a wave-like irregular appearance. At one week after surgery, all surfaces appeared smooth again in the raw (**E**) and segmented (**F**) images. Bars = 250 μm.

During imaging, the subjects were asked to sit in front of the UHR-OCT instrument and look straight ahead to an external central target. To image the structural characteristics and calculate the thickness of all corneal layers including the DEC layer, a 6.1 mm horizontal scan was taken when the light crossed the corneal apex. Custom software was used to process the raw data as described in a previous study [18]. The reflectivity profiles obtained by UHR-OCT were used to measure the thickness of the central cornea and associated sub-layers. The corneal laminae were outlined semi-automatically in the exported images (Figure 1) as we described in a previous study [15]. We imaged each layer at the center of cornea, and we used the software [15] that was designed to outline each layer. Briefly, the software highlighted and locked a point if there was a peak located in the interface. When several points were entered along the surface, the interface was outlined by fitting a curve algorithm to describe the intermediate points. Basically, the software semi-automatically detected and defined each layer of the peak in each A-line. Thus, it could be used even when the cornea was not smooth. The central 100 pixels out of a total of 2,048 pixels, equivalent to 0.298 mm, were used for analysis. 

The repeatability of measuring the thickness of the epithelium and Bowman’s layer has been validated by our previous studies using the same method [15,19], and the thickness of the DEC is similar to Bowman’s layer. Shousha et al. demonstrated the capability of measuring the DEC thickness using UHR-OCT [20]. To evaluate the repeatability of measuring the DEC thickness, 10 subjects were imaged twice before surgery. As proposed by Bland and Altman [21], the coefficient of repeatability (CoR) was expressed as 1.96 × the standard deviation (SD) of the differences between the two measurements. The percentage CoR (CoR%) was calculated as the percentage of CoR divided by the mean of two measurements. The CoR of the DEC in the present study was 1.3 μm (Table 3), the CoR% was 11.5%, and intraclass correlation coefficients (ICC) was 0.883. Thus the repeatability was good.

**Table 3:** Pre- and post-operative thicknesses of the Descemet’s Endothelium Complex, stroma, Bowman’s layer, epithelium, and total cornea.

	Pre-Op (μm)	Post-Op Day 1 (μm)	Post-Op Day 7 (μm)
DEC repeated data (n = 10)**^*†*^**	11.7 ± 1.0		
	11.8 ± 1.1		
DEC	12.7 ± 1.8	17.0 ± 3.3^*^	13.6 ± 3.0
STR	436.1 ± 32.7	461.6 ± 42.8^*^	438.8 ± 30.4
BL	14.4 ± 1.5	14.3 ± 1.5	14.4 ± 1.8
EP	47.4 ± 3.6	49.5 ± 3.7	47.4 ± 4.0
CCT_OCT_	510.3 ± 32.6	542.4 ± 44.4^*^	515.2 ± 31.2
CCT_SM_	503.9 ± 37.8	544.6 ± 48.4^*^	515.7 ± 33.7

Pre-Op, prior to phacoemulsification surgery; Post-Op, following phacoemulsification surgery; DEC, Descemet’s Endothelium Complex; STR, stroma; BL, Bowman’s layer; EP, epithelium; CCT_OCT_, central corneal thickness measured by optical coherence tomography; CCT_SM_, central corneal thickness measured by specular microscopy; *P < 0.05 compared to the pre-operative thickness. **^*†*^**DEC repeated data: to evaluate the repeatability of measuring the DEC thickness, 10 subjects were imaged twice before surgery.

#### Non-contact specular microscopy

A non-contact specular microscope (Topcon SP-3000P Non-contact Specular Microscope, Topcon Corporation, Tokyo, Japan) with an autofocus was used to determine the CCT, the endothelial cell density, and cell shape [22]. After the central endothelium image was obtained, the commercial software automatically marked the cells and calculated the cell density, coefficient of variation (CV) of cell area, percentage of hexagonal cells, and CCT. Due to corneal edema after surgery, it was not possible to automatically count a minimum of 75 cells for each image [23], therefore, at least 50 adjacent endothelial cells at the central area were marked and analyzed. 

#### Non-contact tonometry

Intraocular pressure (IOP) was evaluated by a non-contact tonometer (Full Auto Tonometer TX-F, Canon, Tokyo, Japan). No anesthesia was used during the examination. Three readings were taken and averaged.

### Statistical Analysis

The Statistical Package for the Social Sciences (SPSS 16.0 for Windows XP, Chicago, IL, USA) was used for data analysis. Data were presented as means ± standard deviations. Repeated measurement analysis of variance was used for overall statistical testing. A separate analysis was carried out for each layer of the cornea. Fisher’s Least Significant Difference (LSD) post hoc tests were used to determine if there were differences in thickness of total cornea and corneal sub-layers among all visits. Paired samples t-test was used to compare CCT between instruments at each visit. Changes in endothelial cell density by visit were tested using Wilcoxon test. Spearman correlations among different parameters were determined. P-values < 0.05 were considered statistically significant. Using a similar UHR-OCT instrument, Shousha et al. [20] found that the average DEC thicknesses in a normal young group and a normal elderly group were 10.0 ± 3.0 µm and 16.0 ± 3.0 µm, respectively. The difference between the older group and the young group was 6.0 µm, and we assumed half of the difference (3.0 µm) to calculate the sample size. Based on the 1.3 μm precision of the thickness measurement and the reported DEC thickness of 16.0 ± 2.0 μm in normal elderly [20], the minimum sample size to detect a 3.0 μm group difference with a 99% statistical power was 11. Because the changes in the DEC thickness were expected to be more than 3.0 μm, a sample size of 26 was enough for this study.

## Results

The study included 26 eyes of 26 age-related cataract patients (16 females and 10 males; age: 74.1 ± 5.6 years; range: 60 to 82 years). Pre-operatively, the DEC, stroma, Bowman’s layer, and epithelium were visualized in UHR-OCT images as bands separated by smooth opaque lines (Figure 1). Prior to surgery, the DEC was smooth in appearance (Figure 1A); however, in half of the patients on Day 1 after surgery, it presented an uneven, wave-like appearance (Figure 1C). After one week, the morphologic features of the posterior cornea, including the DEC, had recovered to the pre-surgical appearance (Figure 1E).

**Figure 2:**
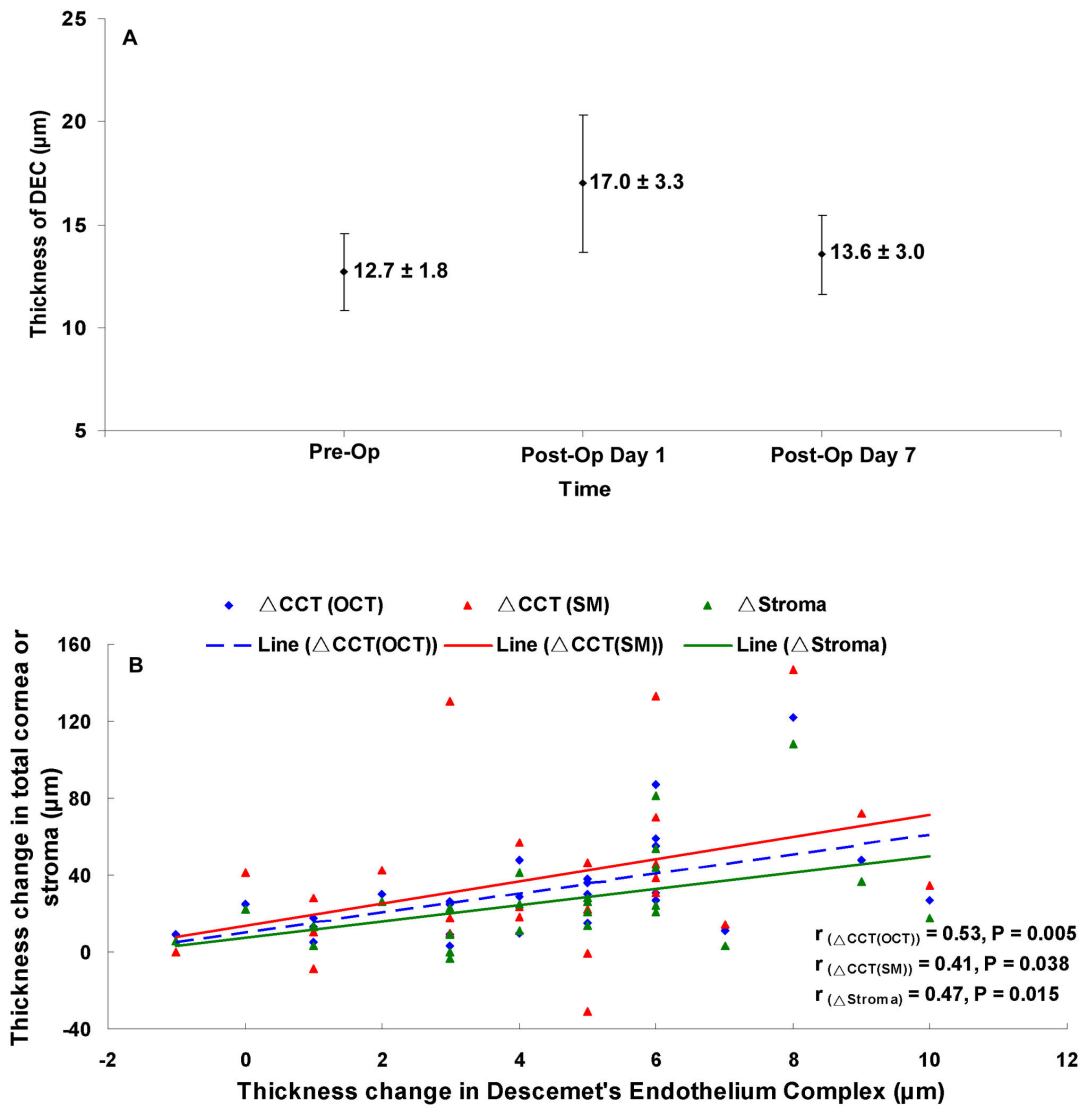
Thickness of the Descemet’s Endothelium Complex (DEC) before and after phacoemulsification surgery. (**A**) Compared to the pre-operative thickness, one day after surgery the DEC was significantly thicker (*, P < 0.05), but by one week it had returned to baseline. (**B**) Correlations between change in the DEC and thickening of the stroma and cornea at post-surgical Day 1. The increased thickness of the central corneal DEC was positively correlated with the thickening of the stroma and central cornea (r range: 0.41 to 0.53, P < 0.05). CCT_(OCT)_, central corneal thickness measured by OCT; CCT_(SM)_, central corneal thickness measured by specular microscopy.

The thickness of the central DEC before phacoemulsification surgery was 12.7 ± 1.8 μm (Table 3, Figure 2A). One day after surgery it was significantly thicker (post hoc, P < 0.001, Table 3, Figure 2A), but by one week it had returned to baseline (P > 0.05, Table 3, Figure 2A). The thickness of the pre-operative stroma was 436.1 ± 32.7 μm (Table 3). One day after surgery it was significantly thicker (post hoc, P < 0.001), and, like the DEC, it recovered to baseline thickness by one week (P > 0.05, Table 3). One day after surgery the increased thickness in the central DEC was positively correlated with the thickening of the stroma and CCT as measured by both UHR-OCT and specular microscopy (r range: 0.41 to 0.53, P range: 0.005 to 0.038, Figure 2B). There was no obvious change in the thickness of Bowman’s layer or the epithelium at any time after the operation (post hoc, P > 0.05, Table 3). The CCT measured by OCT before surgery was 510.3 ± 32.6 μm (Table 3), which was not significantly different from that measured by specular microscopy, 503.9 ± 37.8 μm. By both forms of measurement, the thickness increased on Day 1 after surgery (post hoc, P < 0.001) and then returned to baseline by Day 7 (P > 0.05). 

**Figure 3:**
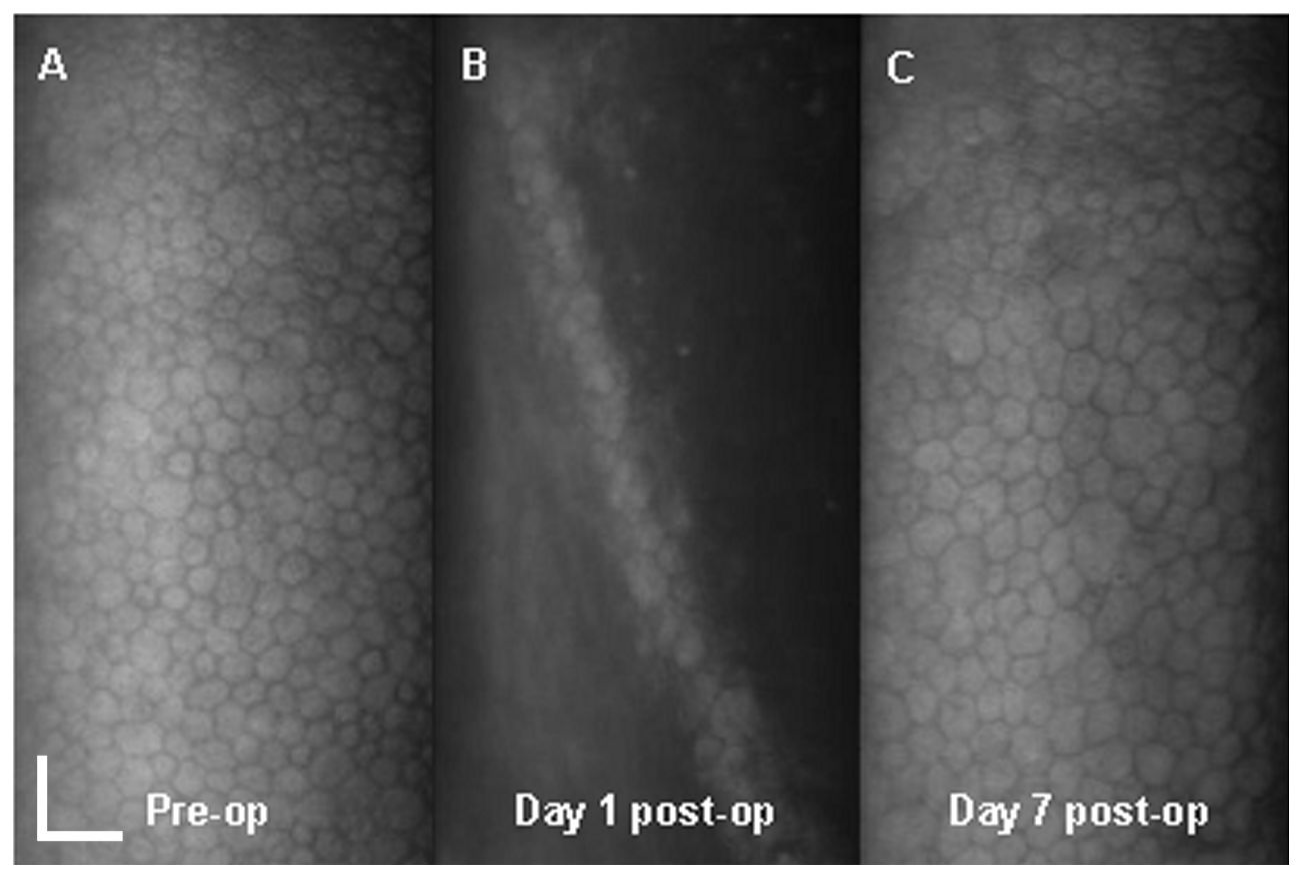
Representative specular microscopy images of endothelial cells before and after cataract surgery. (**A**) Prior to surgery, the endothelial cells were relatively uniform in size and shape. (**B**) Due to corneal edema on Day 1 after surgery, the posterior surface of cornea was uneven, and it was difficult to capture clear images for all of the endothelial cells. The entire endothelial layer was not at the same focus plane, which indicates DEC swelling. A similar finding was also evident in UHR-OCT images (refer to Figure 1C). Nevertheless, the loss of endothelial cells and the increased size of the cells were evident when compared to the pre-operative endothelium. (**C**) The larger cells and decrease in cell density were still evident on Day 7 after surgery. Bars = 50 μm.

The endothelial cell density, determined by non-contact specular microscopy (Figure 3A), in the central cornea before surgery was 2,314.5 ± 288.5 cells/mm^2^ (Table 4). At one day (Figure 3B), there were 5.7% ± 11.7% fewer endothelial cells (P = 0.018). After one week (Figure 3C), there were 8.7% ± 8.2% fewer endothelial cells (P < 0.001). 

**Table 4:** Endothelial cell density and morphology determined by a non-contact specular microscopy before and after cataract surgery.

	Pre-op	1 Day Post-op	1 Week Post-op
Endothelial cell density (cells/mm^2^)	2314.5 ± 288.5	2181.6 ± 376.6^*^	2110.1 ± 347.6^*^
CV of cell area	36.4 ± 6.5	38.3 ± 5.1	36.8 ± 4.5
Percent of hexagonal cells (%)	54.6 ± 7.2	48.7 ± 7.2	52.2 ± 10.0

CV: coefficient of variation.

* P < 0.05, compared with the pre-operative value.

Half of the patients developed wave-like changes in the DECs, and the other half did not. In the patients who developed wave-like changes in the DECs (Group A, Table 5), CCT, stromal thickness, and the DEC thickness increased at 1 day post-operatively compared to the pre-operative level. For the patients that did not develop the wave-like changes in the DECs (Group B, Table 5), only DEC thickness increased at 1 day post-operatively. The other parameters did not increase in thickness compared with baseline. Further, the patients with wave-like changes in DECs had higher increases in DEC thickness and more endothelial cell loss, compared to the ones who did not (Table 5). 

**Table 5:** Comparisons between eyes with and without wave-like changes in DEC.

**Thickness (µm)**	**Group**	**Pre-Op**	**1 Day Post-Op**	**1 Week Post-Op**
DEC	A	13.0 ± 1.9	18.8 ± 3.0^*†^	13.9 ± 2.1
	B	12.4 ± 1.7	15.2 ± 2.7^*^	13.3 ± 1.7
Stroma	A	434.6 ± 28.5	475.6 ± 42.7^*^	439.7 ± 26.6
	B	437.1 ± 37.6	447.5 ± 39.5	440.1 ± 37.6
Bowman’s layer	A	14.5 ± 1.6	14.4 ± 1.5	13.9 ± 1.6
	B	14.3 ± 1.4	14.3 ± 1.6	14.9 ± 1.9
Epithelium	A	47.5 ± 3.1	49.7 ± 2.9	47.3 ± 3.8
	B	47.3 ± 4.1	49.3 ± 4.5	47.4 ± 4.2
CCT _(OCT)_	A	509.6 ± 28.2	558.5 ± 42.1^*^	514.7 ± 24.5
	B	511.1 ± 37.7	526.3 ± 42.2	515.8 ± 37.8
CCT _(SM)_	A	503.9 ± 37.8	544.6 ± 48.4^*††^	515.7 ± 33.7
	B	501.4 ± 44.2	524.5 ± 39.1	513.1 ± 37.7
Percentage of endothelial cell loss (%)	A	-	7.2 ± 16.2	13.0 ± 9.1**^*†††*^**
	B	-	4.1 ± 4.2	4.9 ± 4.3

Group A, wave-like changes in DEC (n = 13); Group B, no wave-like changes in DEC (n = 13); DEC, Descemet’s Endothelial Complex; CCT_(OCT)_, central corneal thickness measured by optical coherence tomography; CCT_(SM)_, central corneal thickness measured by specular microscopy; *, P < 0.05 compared to pre-operative value; **^*†*^**, P = 0.003 compared to Group B. **^*††*^**, P = 0.046 compared to Group B. **^*†††*^**, P = 0.005 compared to Group B.

Mean visual acuity in all subjects improved from 4.85 ± 0.23 at 1 day to 4.90 ± 0.19 at 1 week post-operatively (P = 0.040). The visual acuity at 1 day post-operatively in the patients who showed deformation of the endothelium and who did not was 4.81 ± 0.25, and 4.89 ± 0.18, respectively (P = 0.060). The pre-operative IOP was 12.1 ± 2.3 mmHg, 12.8 ± 3.8 mmHg at 1 day after surgery, and 10.9 ± 2.8 mmHg after one week (LSD post hoc test, P > 0.05 for all comparisons). 

**Figure 4:**
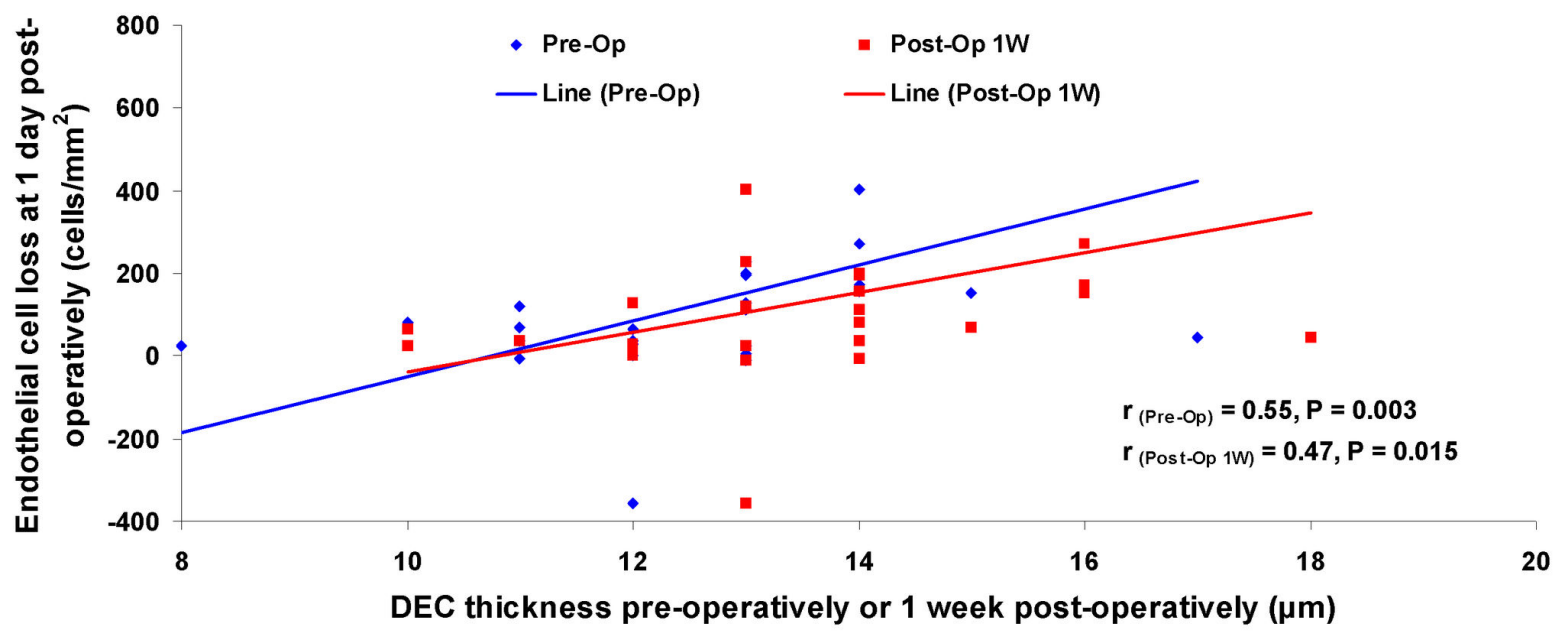
Correlations between the loss of the endothelial cells at post-operative Day 1 and the pre-operative and 1-week post-operative Descemet’s Endothelium Complex (DEC) thickness. The pre-operative and 1-week post-operative DEC thicknesses were positively correlated with the loss of the endothelial cell density at 1 day after surgery (r = 0.55 and 0.47, P = 0.003 and 0.015, respectively).

The pre-operative DEC thickness was positively correlated with the decrease of endothelial cell density between pre-operative and 1 day post-operative values (r = 0.55, P = 0.003, Figure 4, Table 6). The increased thickness of the DEC between the baseline and post-operative Day 1 was negatively correlated with percentage of hexagonal endothelial cells at 1 day and 1 week (r = -0.43 and -0.43, P = 0.029 and 0.030, respectively, Table 6). There were no correlations between age of the patients and baseline DEC thickness or post-operative changes measured by UHR-OCT (P > 0.05). In addition, there were no correlations between age and baseline endothelial cell density or cell loss after surgery (P > 0.05). There was no correlation between endothelial cell density imaged by confocal microscopy at baseline and endothelial cell loss one day after surgery (P > 0.05). Finally, baseline DEC thickness and CCT and the post-operative changes were not correlated with the effective phacoemulsification time, mean energy power, or cataract hardness rating scale (P > 0.05).

**Table 6:** Correlations of the Descemet’s Endothelium Complex (DEC) thickness with other parameters.

DEC thickness (µm)					
Parameter	Pre-Op	1 Day Post-Op	1 Week Post-Op	1 Day Post-Op – Pre-Op	1 W Post-Op – 1 D Post-Op
Endothelial cell loss Post-1D (cells/mm^2^)	r = 0.55, P = 0.003	---	r = 0.47, P = 0.015	---	---
Difference of CCT (µm)	---	r = 0.53, P = 0.005	---	---	---
Difference of stromal thickness (µm)	---	r = 0.42, P = 0.036	---	---	---
Percentage of hexagonal cells at 1 Day (%)	---	---	---	r = -0.43, P = 0.029	r = -0.53, P = 0.006
Percentage of hexagonal cells at 1 Week (%)	---	r = -0.41, P = 0.038	---	r = -0.43, P = 0.030	---

Only values where P < 0.05 are shown. Differences of CCT and of stromal thickness were based on pre-operative and 1 day post-operative measurements. DEC, Descemet’s Endothelial Complex; CCT, central corneal thickness.

## Discussion

The cornea consists of five layers: epithelium, Bowman’s layer, stroma, Descemet’s membrane, and endothelium. The stroma has a tendency to swell under normal physiological conditions. This swelling is prevented by active fluid pumping of the endothelium, thereby assuring maintenance of normal thickness and transparency of the cornea [3]. Phacoemulsification may influence the barrier function of the endothelium due to endothelial cell loss during surgery [7]. Morphological evaluation of the endothelium is a sensitive way to assess changes resulting from surgical trauma. UHR-OCT is a validated technique to visualize the DEC, stroma, Bowman’s layer, and the epithelium [16,20,24]. 

To the best of our knowledge, this is the first report of in vivo changes in corneal sub-layer thicknesses in patients after cataract surgery. Our data showed that the CCT increased one day after surgery and recovered by one week after surgery. This is in agreement with Bolz et al. [25] and indicates that corneal swelling is a very common but transient phenomenon after phacoemulsification [2]. Within the corneas, the thicknesses of the DEC and stroma were increased on the first post-surgical day but returned to baseline one week after surgery. While the percent increase in the DEC thickness, 33.9%, was greater than for the stroma, 5.8%, most of the increase in corneal thickness was attributed to the stroma, which became 25.5 µm thicker. The thicknesses of the epithelium and Bowman’s layer did not change during the early post-operative period. Thus, the increase of CCT was mainly due to the increase in the stromal thickness. Although OCT measurement may be affected by the changes in corneal refractive index due to corneal edema after surgery, it has been suggested that potential errors of estimating the thickness induced by edema could be below 3.0% [26]. The possible error may not explain measured swelling of the cornea and DEC in the present study. 

The reason for the different thickness changes in the corneal sub-layers may be due to uneven resistance to water flow through the entire cornea. The corneal stroma has a tendency to absorb water, while the endothelium, with its pumping of electrolytes and water from the tissue to the anterior chamber, is about 220 times more resistant to water absorption than the corneal stroma [27]. Thus impairment to the endothelial cells and/or their pumps during intraocular surgery results in stromal absorption of fluid and tissue swelling. The swelling pressure can be 50.0 to 60.0 mmHg in all directions at the same time [22]. The tightly packed anterior stroma has been proposed to be less affected than the posterior stroma when the cornea swells [28]. Since the stronger, more rigid regions of the cornea are located anteriorly and peripherally [28,29], it is the DEC rather than the epithelium or Bowman’s layer that bulges toward the anterior chamber. Therefore, we noted that the amount of edematous thickening of the DEC was positively correlated with the stromal thickening. Compared to the stroma, the expansion of the DEC was much smaller due to the relative thinness of the DEC. Descemet’s membrane is a basement membrane [11] and wouldn’t be expected to swell; therefore we postulate that the endothelial cells became swollen. In some corneas, when the pressure of stromal swelling was sufficiently high, it caused the deformation of the DEC. This DEC deformation may have relieved the high pressure of the stroma and preserved corneal function. Additionally, the exact location of distortion in the DEC may be related to the location of damaged endothelial cells. Because the center of the cornea is weaker than periphery [29], there is a greater likelihood that deformation of the DEC would occur at the center. 

The transient post-operative corneal swelling that commonly occurs after phacoemulsification is probably the result of damage to endothelial cells by the surgery [25]. Many factors can cause endothelial injury during cataract surgery, such as direct mechanical trauma, ultrasound energy, and the irrigating solution [2]. Endothelial cells are non-replicative, and consequently the damaged area is covered by incomplete enlargement and migration of residual cells [3]. One day after surgery, specular microscopy showed that the endothelial cell density was lower, and the cell population consisted of cells of many different sizes and irregular shapes. These corneal insults can lead to transient post-operative corneal swelling after most cataract procedures, albeit sometimes at a subclinical level. Although Shousha et al. [20] reported that the DEC thickness increased in normal elderly group compared with normal young group, these changes with aging may not necessarily correlate with clinically relevant loss of endothelial cells. Using UHR-OCT, we observed that the morphology of the DEC in some cases appeared as irregular, wave-like lines, probably as a result of excessive edema. There was no significant difference in IOP before and after surgery in the present study. Therefore, IOP probably does not play a role in changes of the DEC. 

Similar to other studies [30-32], the endothelial cell density one week after surgery in this study was decreased and endothelial cell loss was evident. In spite of this decrease in cell density, both the smooth appearance and thickness of the endothelial layer at one week after surgery had recovered to the pre-surgical level. Based on our data and other studies [7], it appears that a healthy cornea can recover rapidly from transient increases in the CCT after phacoemulsification. Thus, when the numerical density of the corneal endothelial cells is within the physiological threshold, partial loss of these cells does not compromise the pumping activity as a whole. However, when the density is low (below 600 to 800 cells/mm^2^), corneal decompensation, bullous keratopathy, and loss of vision occurs [4]. Although specular microscopy provides valuable clinical information, it may not able to predict mild to moderate cornea edema progression after cataract surgery. 

Establishing the relationship between the thickness and en-face morphology of the DEC may help to better understand the corneal responses to surgical procedures that can cause subclinical wounding during phacoemulsification. The pre-operative DEC thickness was positively correlated with the decrease of endothelial cell density at 1 day after surgery. However, the relationship between endothelial cell density imaged by confocal microscopy at baseline and endothelial cell loss one day after surgery was not evident in the present study. Our data showed that morphological and thickness analysis of the DEC obtained by UHR-OCT provided a more precise indicator of endothelial cell damage than specular microscopy alone. The cross-sectional thickness of the DEC pre-operatively imaged by UHR-OCT may indicate the integrity of the endothelium. In turn, this information may be used for predicting the endothelial cell loss after phacoemulsification. For the patients who did not develop wave-like morphological changes, the DEC increased after surgery, but stromal and total corneal thicknesses did not change. This indicates that the DEC may be a more sensitive parameter than other thickness parameters. It may be not feasible for everyone to use UHR-OCT to image the DEC due to the high cost and limited availability of commercial UHR-OCT instruments. Measuring stromal or total corneal thickness may be valuable in roughly determining corneal response to surgery. Considering the high resolution and wide scan width, UHR-OCT may add an alternative modality for studying the DEC thickness and morphology; however, UHR-OCT may not replace specular microscopy for imaging the endothelial cells. 

The values for CCT estimated by UHR-OCT and specular microscopy were similar to one another. The thickness of epithelium in the present study, 47.4 ± 3.6 µm, is in agreement with previously reported values [33]. However, the thicknesses of both the DEC and Bowman’s layer were slightly thinner than reported in other studies using UHR-OCT [16,20]. These differences might be attributed to subjects of different races and regions.

There were some limitations in this study. The follow-up time was only one week after surgery. Although we found that the corneal sub-layer thicknesses recovered post-operatively, other parameters such as corneal endothelial cell density did not return to baseline. It is possible that a longer follow-up period would have shown that the endothelial cell density tended to recover or possibly continued to decrease [30]. A second limitation is that we focused only on the changes at the central DEC. Further studies are warranted to detect changes in the mid-peripheral and peripheral regions, especially the region near the incision. 

In summary, one day after phacoemulsification in age-related cataract patients, the cornea became thickened principally due to stromal edema and to a lesser extent DEC edema. The DEC displayed different types of morphological changes. The complex sub-layer deformations and corneal edema recovered by one week after surgery. This indicated that endothelial cells, though less dense than before surgery, were able to recover the functional state and restore the normal thickness of cornea. The thicknesses of the epithelium and Bowman’s layer did not change during the early post-operative period. The cross-sectional thickness of the DEC pre-operatively imaged by UHR-OCT may indicate the integrity of the endothelium, which, in turn, may be used for predicting endothelial cell loss after phacoemulsification. Considering the high resolution and wide scan width, UHR-OCT may add an alternative modality for studying the DEC thickness and morphology, although UHR-OCT cannot replace specular microscopy in imaging the endothelial cells. 
